# Renal denervation for atrial fibrillation: a comprehensive updated systematic review and meta-analysis

**DOI:** 10.1038/s41371-022-00658-0

**Published:** 2022-01-29

**Authors:** Khaled Nawar, Ahmed Mohammad, Edward J. Johns, Mohammed H. Abdulla

**Affiliations:** 1 School of Medicine, University College Cork, Cork, Ireland; 2 Department of Physiology, University College Cork, Cork, Ireland

**Keywords:** Atrial fibrillation, Clinical trials

## Abstract

The study aims to compare clinical outcomes following renal denervation (RDN) in hypertensive patients with atrial fibrillation (AF). Three online databases were searched (MEDLINE, EMBASE and PubMed) for literature related to outcomes of RDN on hypertension and AF, between January 1, 2010, and June 1, 2021. Where possible, risk ratios (RR) and mean differences (MD) were combined using a random effects model. Significance was set at *p* ≤ 0.05. Seven trials were included that assessed the effect of adding RDN to pulmonary vein isolation (PVI) in patients with hypertension and AF. A total of 711 patients (329 undergoing PVI + RDN and 382 undergoing PVI alone), with an age range of 56 ± 6 to 68 ± 9 years, were included. Pooled analysis showed a significant lowering of AF recurrence in the PVI + RDN (31.3%) group compared to the PVI-only (52.9%) group (*p* < 0.00001). Pooled analysis of patients with resistant hypertension showed a significant mean reduction of systolic blood pressure (SBP) (−9.42 mm Hg, *p* = 0.05), but not diastolic blood pressure (DBP) (−4.11 mm Hg, *p* = 0.16) in favor of PVI + RDN. Additionally, the pooled analysis showed that PVI + RDN significantly improved estimated glomerular filtration rate (eGFR) (+10.2 mL/min per 1.73 m^2^, *p* < 0.001) compared to PVI alone. RDN procedures in these trials have proven to be both safe and efficacious with an overall complication rate of 6.32%. Combined PVI and RDN is beneficial for patients with hypertension and AF. Combined therapy showed improvement in SBP and eGFR, reducing the risk of AF recurrence. RDN may serve as an innovative intervention in the treatment of AF.

## Introduction

Atrial fibrillation (AF) is the most common type of heart arrhythmia currently affecting 0.51% of the population globally [1]. The prevalence of AF has increased by 33% over the last 20 years particularly due to the increase in the ageing population [1, 2]. In addition to the high prevalence of AF, the spectrum and severity of the condition varies tremendously. However, a common underpinning in AF patients is that hypertension (HTN) is associated with one in five cases of AF [3].

Despite the availability of a variety of pharmacological and lifestyle interventions, around 50% of patient with HTN remain resistant to such strategies [4]. This highlights the existence of a more complex pathophysiological mechanism that defies current therapeutic regimens [5]. More recently, the development of endovascular catheters has allowed for easy access to the renal artery lumen to specifically ablate renal nerves and hence multiple trials were executed over the last decade to carefully examine the effect on renal sympathetic outflow and the downstream effect on blood pressure [6]. The benefits as such of renal denervation (RDN) were further reiterated in multiple trials and a recent network meta-analysis of 20 trials (*n* = 2152) showed that RDN of main renal artery branches in addition to antihypertensive therapy is most effective in reducing office blood pressure and that RDN using this approach was superior in reducing ambulatory blood pressure compared to sham or antihypertensive therapy alone [6].

Catheter ablation through pulmonary vein isolation (PVI) in patients who fail to demonstrate a reduction in AF recurrence following pharmacological agents is currently a highly effective intervention [7]. Despite PVI being superior to drug therapy, the intervention shows a failure rate of 20–50%, which warrants further investigation of alternative strategies for treating AF [8, 9]. The pathophysiological association between an elevated sympathetic tone, AF and HTN, in addition to the significant failure rate of PVI, prompted the investigation of the effect of RDN on AF and hence, a pilot trial was executed [10]. The trial demonstrated the superiority of combining RDN and PVI and their additive effect in reducing both blood pressure and AF recurrence [10]. Subsequently, multiple clinical trials investigated the efficacy of RDN in addition to PVI to lower AF recurrence. To this end, this analysis aimed to analyse the published literature to compare the effect of RDN and PVI on AF recurrence, blood pressure and estimated glomerular filtration rate (eGFR) in hypertensive patients. Secondarily, the study aimed to examine the overall safety of the combined techniques.

## Materials and methods

This study utilised the Preferred Reporting Items for Systematic Reviews and Meta-Analyses (PRISMA) guidelines and Revised Assessment of Multiple Systematic Reviews guidelines to perform and design the review [11, 12]. This included using an a priori study design; exhaustive literature search; duplication of study screening, selection and data extraction; scientific quality and bias assessment of included studies; reporting of study characteristics and utilising appropriate statistical methods for assessment of study findings [11, 12].

### Literature search and inclusion criteria

Two authors searched three online databases (MEDLINE, PubMed, and Embase) for papers published from January 1, 2009, to June 1, 2021, using the following combination of keywords: RDN, renal sympathetic denervation, catheter-based RDN, kidney denervation, renal artery denervation. Studies that were retrieved from the initial database search were published in English and from human trials. Additionally, any missed studies were included into screening following a full reference screen of relevant studies. The inclusion criteria were as follows: (1) Original research articles, (2) published after January 1, 2009, in English language, (3) Level I or Level II prospective comparative studies that (4) assessed the effect of RDN on AF in patients with essential HTN that are undergoing PVI. The exclusion criteria were as follows: (1) Studies that assessed patients with secondary HTN, (2) type I diabetes mellitus, (3) late-stage kidney disease/failure (mean eGFR <45 mL/min per 1.73 m^2^), (4) congestive heart failure, (5) left-ventricular ejection fraction (LVEF) < 35% (6) studies published in non-English language.

### Literature screening

**Fig. 1:**
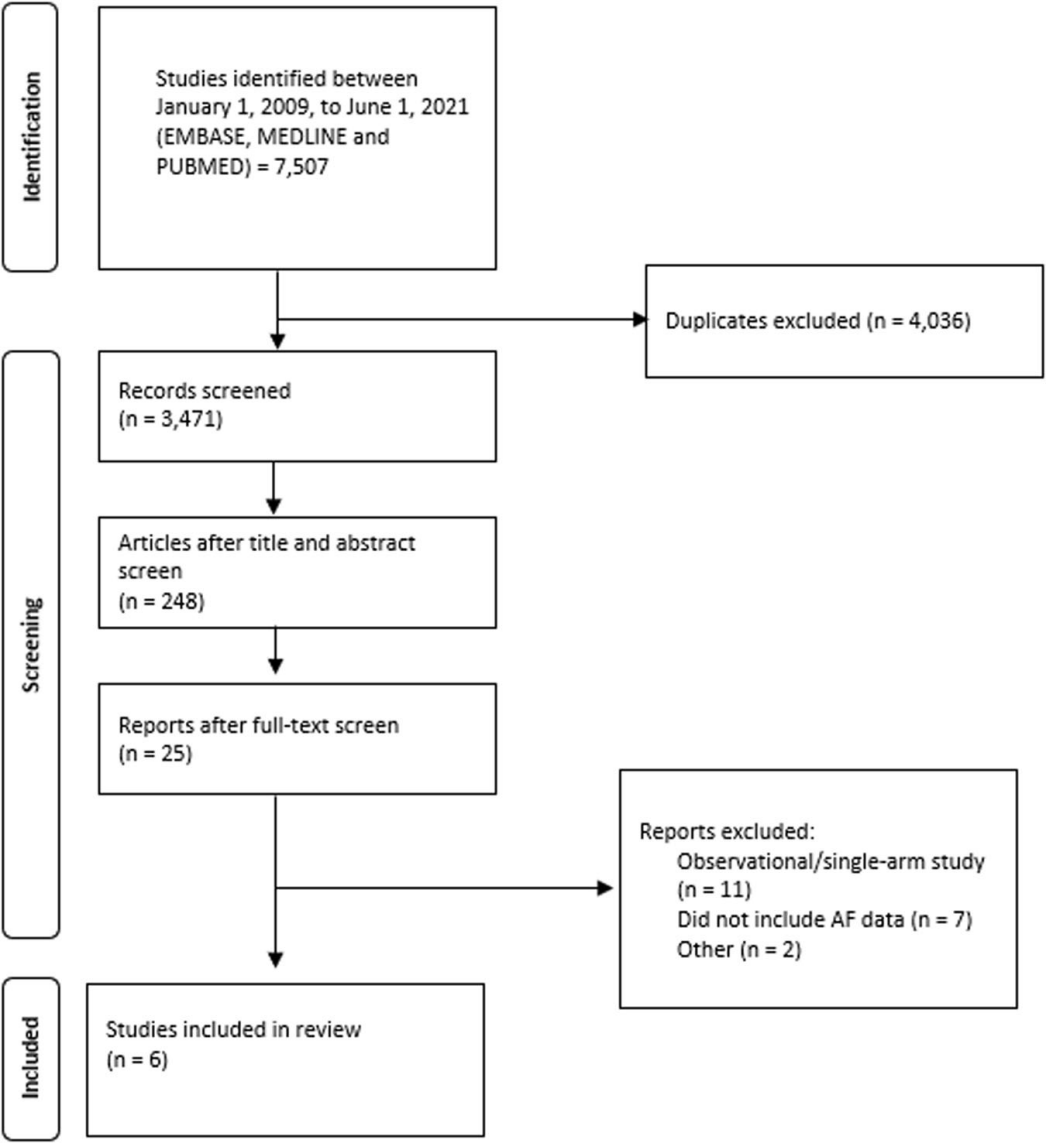
PRISMA chart. Flow chart outlining the screening process for the included/excluded studies and detailing the results following each screening stage.

The studies were screened during the three stages (title, abstract and full-text screen) independently and in duplicates by two authors (KN and AM). Disagreements were internally discussed before moving to the subsequent stage of screening. A PRISMA flow chart of the literature screening is shown in Fig. 1 [12].

### Quality assessment of included studies

The Cochrane risk of bias (ROB) tool was used to assess quality and publication bias of the individual studies that were randomized (The Cochrane Collaboration, Copenhagen, Denmark) (Supplementary Fig. 1). Studies that were non-randomized but prospective were assessed for quality and publication bias using The Methodological Index for Non-Randomized Studies (MINORS) [13] (Supplementary Table 1).

### Interviewer agreement

The Kappa (k) scores were used at each stage of the screening process in order to determine inter-rater reliability as well as agreement [14]. The k scores were all above the 0.6 threshold which indicates strong inter-rater reliability [14].

### Data extraction

Two authors (KN and AM) independently collected and extracted data into a standardized form, in Excel 2019 (Microsoft, Redmond, WA, USA). The following information, if available, was extracted from the studies: primary author and year, study design and purpose, sample size, age and gender information, country of study, follow-up time frame, baseline study sample characteristics (number of HTN medications, eGFR, presence of type II diabetes, coronary artery disease (CAD), LVEF, and left-atrial diameter (LAD), AF type, and systolic and diastolic blood pressure), follow-up data on AF recurrence and blood pressure, and safety complications.

### Data analysis

This analysis primarily aimed to compare the effects of RDN on AF recurrence in hypertensive patients that were undergoing PVI for treatment of AF. The main outcomes were AF recurrence, the blood pressure lowering effect of RDN as well as the effect of RDN of eGFR. AF recurrence was defined as episodes of atrial tachyarrhythmias lasting >30 s during the follow-up period. Secondarily, the analysis aimed to assess the safety, efficacy, and sustainability of RDN.

The pooled analysis of quantitative results was synthesized using RevMan 5.3 (The Cochrane Collaboration, Copenhagen, Denmark). Pooling of data was conducted using a random effects model and study weighing was based on inverse variance. A minimum of three studies was required for outcome pooling. To assess for heterogeneity due to differences in study methods and populations, the *I*^2^ values were used [15]. Moreover, a sensitivity analysis was performed in analyses showing a high heterogeneity of *I*^2^ > 65% by removing each study separately and examining the effect on the *I*^2^ value [16].

Treatment effects for dichotomous variables were compared using risk ratios (RR) and 95% confidence intervals (CI), and those for continuous variables were compared using mean difference (MD) and 95% CI. For studies that did not report standard deviations (SD), the values were estimated according to the guidelines outlined in the *Cochrane Handbook for Systematic Reviews* [16].

## Results

### Literature screen and baseline study characteristics

Figure 1 is a PRISMA chart demonstrating the results of the literature search. Table 1 outlines the characteristics of the six included studies [17–22]. Table 2 summarizes the inclusion criteria, as well as the procedural methods employed by the studies.

**Table 1:** Baseline characteristics of included studies.

Study Author (Year)	Turagam-HFIB 2 (2021) [22]		Turagam-HFIB 1 (2021) [22]		Steinberg et al., (2020) [21]		Kiuchi et al.,(2018) [20]		Kiuchi et al., (2017) [19]		Kiuchi et al., (2016) [18]		Pokushalov et al., (2014) [17]	
**Study design**	RCT		RCT		RCT		RCT		Prospective Non-randomized		RCT		RCT	
**Country**	USA		USA		Germany, Poland, and Russia		Brazil		Brazil		Brazil		USA, Russia	
**F/U period (months)**	24		24		12		12		22.4 ± 4.10		12		12	
**Study groups**	**PVI** + **RDN**	**PVI**	**PVI** + **RDN**	**PVI**	**PVI** + **RDN**	**PVI**	**PVI** + **RDN**	**PVI** + **Spironolactone**	**PVI** + **RDN**	**PVI**	**PVI** + **RDN**	**PVI**	**PVI** + **RDN**	**PVI**
**Number of patients**	28	22	13	17	154	148	33	36	39	96	21	24	41	39
**Age**	64.0 ± 7.00	65.0 ± 8.00	59.0 ± 10.0	68.0 ± 9.00	59 (IQR 54–65)^a^	60 (IQR 58–65)^a^	56.8 ± 6.50	58.4 ± 5.10	60.0 ± 14	59.0 ± 15.0	68.0 ± 9.0	66.0 ± 9.0	56.0 ± 6.00	56.0 ± 6.00
**Number of females**	12	8	5	8	63	57	8	6	15	31	8	8	10	15
**Number anti-HTN drugs**	2.80 ± 0.95	2.50 ± 1.00	2.80 ± 1.20	2.80 ± 1.30	2.10^b^	2.10^b^	3.50 ± 0.50	3.74 ± 0.40	2.20 ± 0.40	2.40 ± 0.50	3.41 ± 0.60	3.30 ± 0.50	3.40 ± 0.70	3.40 ± 0.80
**% Patients with T2D**	17.9	36.4	0.00	17.6	10.4	12.2	24.2	27.8	35.9	37.5	76.2	54.2	12.2	22.5
**% Patients with CAD**	NR	NR	NR	NR	9.10	6.80	15.2	25.0	NR	NR	57.1	58.3	12.2	10.3
**LAD (mm)**	54.0 ± 0.90	47.0 ± 1.30	51.0 ± 0.90	46.0 ± 0.70	48.00 (CI 47.0,49.0)^c^	48.0 (CI 46.0,49.0)^c^	NR	NR	NR	NR	54.1 ± 3.20	44.9 ± 3.90	47.0 ± 5.00	47.0 ± 4.00
**LVEF (%)**	62.0 ± 6.00	64.0 ± 5.00	60.0 ± 6.00	61.0 ± 5.00	62.0 ± 5.00	62.0 ± 5.00	62.2 ± 7.20	61.2 ± 5.70	65.8 ± 12.8	66.5 ± 10.0	62.7 ± 6.60	63.5 ± 6.80	60.0 ± 4.00	61.0 ± 5.00

Data for age, antihypertensive medications, left-atrial diameter, and left-ventricular ejection fraction are displayed as means and standard deviation (SD).

*RCT* randomized control trial, *F/U* follow-up, *PVI* pulmonary vein isolation, *RDN* renal denervation, *IQR* interquartile range, *CI* confidence interval, *HTN* hypertension, *T2D* type II diabetes, *CAD* coronary artery disease, *LAD* left-atrial diameter, *NR* not reported, *LVEF* left-ventricular ejection fraction.

^a^This study reported data as median and interquartile range.

^b^Standard deviation could not be estimated for this study.

^c^This study reported data as mean and confidence interval.

**Table 2:** Criteria and intervention method of included studies.

Study Author (Year)	Turagam-HFIB 2 (2021) [22]	Turagam-HFIB 1 (2021) [22]	Steinberg et al., (2020) [21]	Kiuchi et al., (2018) [20]	Kiuchi et al., (2017) [19]	Kiuchi et al., (2016) [18]	Pokushalov et al., (2014) [17]
**Study population**	• Paroxysmal and persistent AF • Drug-resistant HTN (Office SBP ≥ 160 or DBP ≥ 100) • At least 1 antihypertensive • eGFR>45	• Paroxysmal and persistent AF • Drug-resistant HTN (Office SBP ≥ 160 or DBP ≥ 100) • At least 1 antihypertensive • eGFR>45	• Paroxysmal AF • Drug-resistant HTN (Office SBP ≥ 130 or DBP ≥ 80) • At least 1 antihypertensive	• Paroxysmal AF or symptomatic refractory AF • Drug-resistant HTN (ASBP ≥ 130, ADBP ≥ 80) • At least 3 antihypertensive • eGFR ≥60 and microalbuminuria	• Paroxysmal AF • Drug-controlled HTN (130>ASBP ≥ 100) • eGFR >15 (if eGFR>60 have microalbuminuria)	• Refractory paroxysmal or persistent AF • Drug-controlled HTN (130>ASBP ≥ 100) • eGFR between 30–89 and if >60 microalbuminuria	• Refractory paroxysmal or persistent AF • Moderate drug-resistant HTN (Office BP ≥ 140/90) or severe drug-resistant HTN (Office BP ≥ 160/100) • At least 3 antihypertensive (including 1 diuretic) • eGFR ≥ 45
**AF definition**	Persistent AF = AF ≥ 7 Days	Persistent AF = AF ≥ 7 Days	PAF = AF up to 7 days	PAF = AF up to 7 days	PAF = AF up to 7 days	PAF = AF up to 7 days Persistent AF = AF ≥ 7 Days	PAF = AF up to 7 days Persistent AF = AF ≥ 7 Days
**% PAF**	70.0	66.7	100.0	100.0	100.0	60.0	43.8
**% Persistent AF**	30.0	33.3	0.00	0.00	0.00	40.0	56.2
**PVI method**	Radiofrequency ablation	Radiofrequency ablation	Cryoballoon catheter	Radiofrequency ablation	Radiofrequency ablation	Radiofrequency ablation	Radiofrequency ablation
**RDN method**	Vessix	ThermoCool	Irrigated tip and RDN catheter	EngligHTN	Irrigated tip	Irrigated tip	ThermoCool (*n* = 20), Symplicity (*n* = 21)

*HTN* hypertension, *AF* atrial fibrillation, *SBP* systolic blood pressure, *DBP* diastolic blood pressure, *ASBP* ambulatory systolic blood pressure, *ADBP* ambulatory diastolic blood pressure, *eGFR* estimated glomerular filtration rate, *BP* blood pressure, *PAF* paroxysmal atrial fibrillation, *PVI* pulmonary vein isolation, *RDN* renal denervation.

Units for blood pressure and eGFR are in mm Hg and mL/min per 1.73 m^2^ respectively.

The seven trials included a total of 711 patients, with 329 patients in the PVI + RDN group and 382 patients in the PVI-only group (Table 1). The mean age ranged between 56 ± 6 and 68 ± 9 years and a follow-up time ranging from 12 to 24 months (Table 1). There was a total of 623 patients with paroxysmal AF and 88 patients with persistent AF (Table 2).

### Effect of RDN on AF recurrence

**Fig. 2:**
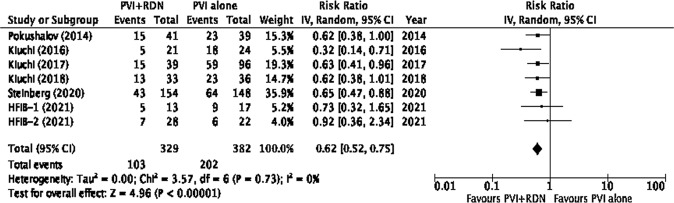
Effects of renal denervation on atrial fibrillation. Forest plot of the pooled comparison between PVI + RDN and PVI alone in the rate of AF recurrence of all included studies. IV inverse variance, df degrees of freedom.

All the included studies reported the rate of AF recurrence throughout the follow-up period (Table 3). The rate of AF recurrence was significantly lower across five trials in the RDN + PVI group [17–21]. Although the rates of AF recurrence were lower in the PVI + RDN groups of HFIB-1 and HFIB-2 trials, they failed to show a significant difference [22]. The overall pooled results of the recurrence of AF demonstrated a significantly lower rate of AF recurrence in the PVI + RDN (31.3%) group compared to the PVI-only (52.9%) group (*p* < 0.00001) (Fig. 2).

**Table 3:** Atrial Fibrillation recurrence following interventions and baseline and 12-month follow-up blood pressure.

Study Author (Year)	Study group	% AF recurrence at follow-up	Baseline SBP (mm Hg)	SBP at follow-up (mm Hg)	Mean difference (mm Hg)	Baseline DBP (mm Hg)	DBP at follow-up (mm Hg)	Mean difference (mm Hg)
**Turagam-HFIB 2 (2021)**^**a**^ [22]	PVI + RDN	25.0	146.6 ± 20.6	138.2	−8.40 ± 25.1	81.4 ± 13.4	82.6	1.20 ± 12.4
	PVI	27.3	143.4 ± 18.4	142.8	−0.60 ± 27.2	79.1 ± 12.4	80.8	1.70 ± 11.1
	*P*-value	NS	NS	___	NS	NS	___	NS
**Turagam-HFIB 1 (2021)**^**a**^ [22]	PVI + RDN	38.5	147.0 ± 31.0	152.3	5.30 ± 25.8	84.1 ± 25.0	84.7	0.630 ± 14.7
	PVI	52.9	153.0 ± 20.0	144.4	−8.60 ± 24.1	88.0 ± 12.0	82.5	−5.50 ± 12.9
	*P*-value	NS	NS	___	NS	NS	___	NS
**Steinberg et al., (2020)** [21]	PVI + RDN	27.9	150.0 ± 9.50	135.0 ± 9.50	−16.0 ± 12.663	90.0 ± 6.33	79.0 ± 9.50	−11.0 ± 9.50
	PVI	43.2	151.0 ± 9.31	147.0 ± 9.31	−3.00 ± 15.5	90.0 ± 9.31	88.0 ± 9.31	−2.00 ± 15.5
	*P*-value	0.006	NS	___	<0.0001	NS	___	<0.0001
**Kiuchi et al., (2018)**^**b**^ [20]	PVI + RDN	39.4	142.0 ± 6.00	123.0 ± 4.00	−19.0 ± 6.83	103.0 ± 8.00	82.0 ± 4	−21.0 ± 8.54
	PVI	63.9	140.0 ± 6.00	130.0 ± 6.00	−10.0 ± -8.76	103.0 ± 7.00	89.0 ± 5.00	−14.0 ± 10.1
	*P*-value	0.043	NS	___	<0.0001	NS	___	NS
**Kiuchi et al., (2017)**^**b**^ [19]	PVI + RDN	38.5	Controlled HTN	Controlled HTN	___	Controlled HTN	Controlled HTN	___
	PVI	61.5	Controlled HTN	Controlled HTN	___	Controlled HTN	Controlled HTN	___
	*P*-value	0.015	___	___	___	___	___	___
**Kiuchi et al., (2016)**^**b**^ [18]	PVI + RDN	23.8	Controlled HTN	Controlled HTN	___	Controlled HTN	Controlled HTN	___
	PVI	75.0	Controlled HTN	Controlled HTN	___	Controlled HTN	Controlled HTN	___
	*P*-value	0.001	___	___	___	___	___	___
**Pokushalov et al., (2014)** [17]	PVI + RDN	36.6	163.0 ± 18.0	142.0 ± 11.0	−21.0 ± 20.0	89.0 ± 11.0	79.0 ± 5.00	−10.0 ± 11.6
	PVI	59.0	164.0 ± 17.0	162.0 ± 10.0	−2.00 ± 22.8	88.0 ± 11.0	86.0 ± 5.00	−2.00 ± 13.7
	*P*-value	0.046	NS	___	0.0002	NS	___	0.006

Data for blood pressures are displayed as means and standard deviation (SD).

*PVI* pulmonary vein isolation, *RDN* renal denervation, *AF* atrial fibrillation, *SBP* systolic blood pressure, *DBP* diastolic blood pressure, *NS* not significant.

^a^Blood pressure data from these studies was at 12-month follow-up for comparison purposes.

^b^These studies reported ambulatory blood pressure data.

### Effects of RDN on blood pressure

**Fig. 3:**
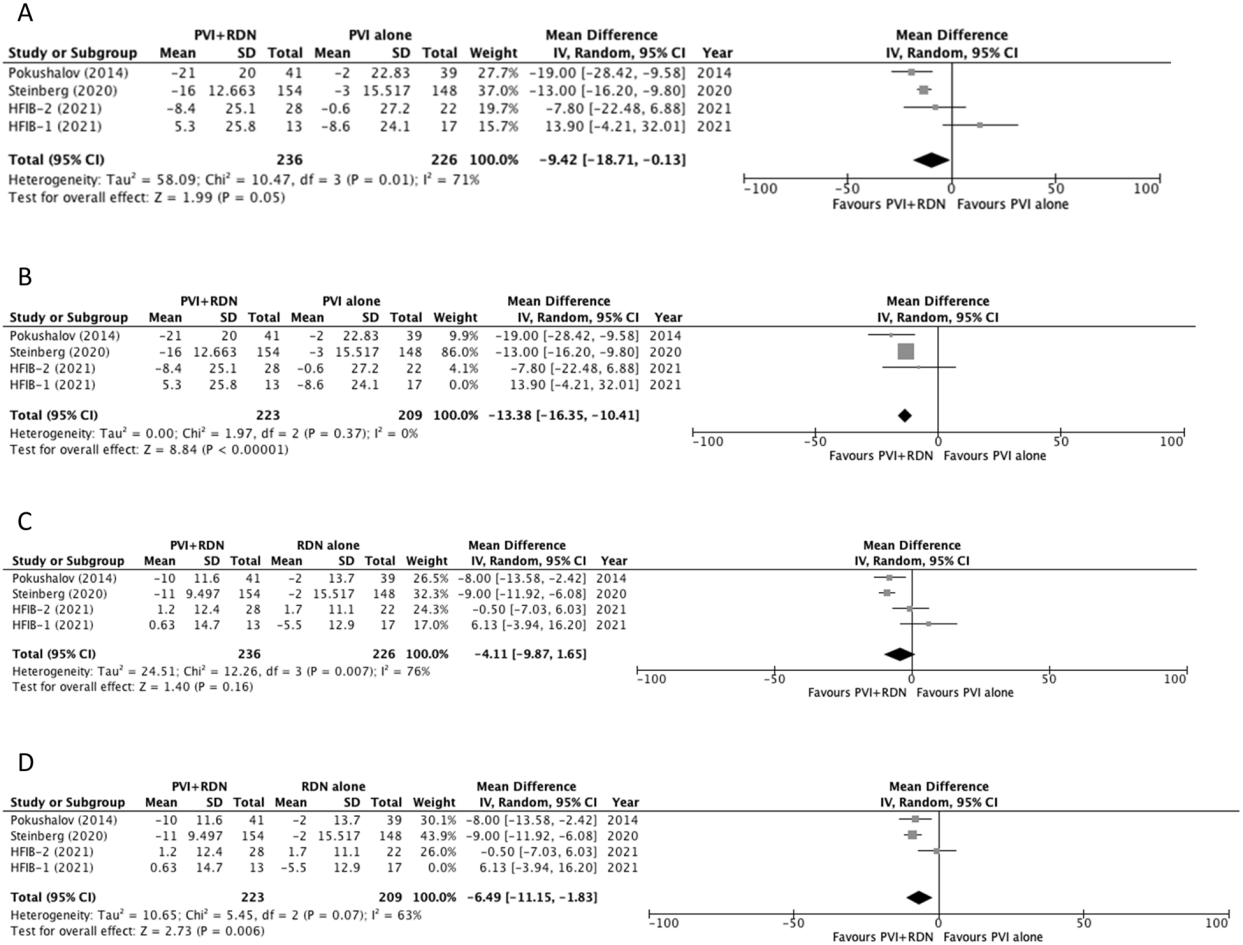
Effects of renal denervation on blood pressure. Forest plot of pooled comparison of office BP between PVI + RDN and PVI (**A**) SBP (**B**) SBP sensitivity analysis after HFIB-1 removal (**C**) DBP (**D**) DBP sensitivity analysis after HFIB-1 removal. IV inverse variance, df degrees of freedom.

All included studies reported follow-up data on blood pressure following the procedure (Table 3). Two of the studies included patients with drug-controlled HTN and one reported blood pressure as ambulatory rather than office and thus were excluded from the meta-analysis [18–20]. For the pooled analysis, 12-month follow-up data was used to compare changes in blood pressure. Two studies reported a significant reduction in both office systolic blood pressure (SBP) and diastolic blood pressure (DBP) in the PVI + RDN group vs. the PVI alone group [17, 21]. One study reported a significant reduction in ambulatory SBP in the PVI + RDN group vs. the PVI alone group [20]. When pooled, the overall results showed a significant MD in SBP of −9.42 mm Hg in the PVI + RDN group vs. the PVI alone group (*p* = 0.05) (Fig. 3A). The pooled DBP analysis failed to show a significant difference between the groups (*p* = 0.16) (Fig. 3C). The pooled analysis for both SBP and DBP showed a high heterogeneity of *I*^2^ value = 71% and 76% respectively.

A sensitivity analysis was conducted by removing each study consecutively and assessing its effect on the SBP and DBP pooled analyses (Fig. 3B,D). Following the removal of HFIB-1, the results indicated a more strongly significant difference of *p* < 0.00001 for SBP and a significant difference of *p* = 0.006 for DBP demonstrating the superiority of the blood pressure lowering effect of the PVI + RDN group and the heterogeneity for both SBP and DBP decreased to *I*^2^ to 0% and 63% respectively (Fig. 3B, D).

### Effect of RDN on eGFR

**Fig. 4:**
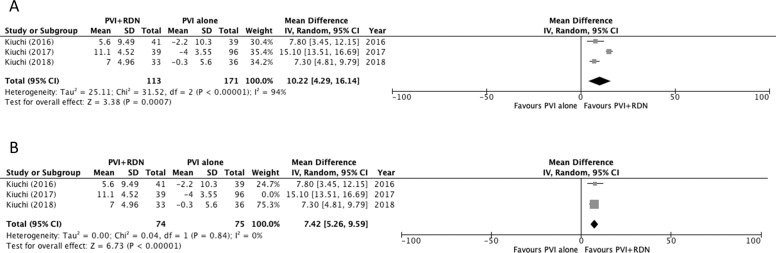
Effects of renal denervation on eGFR. Forest plot of **A** pooled comparison of eGFR between PVI + RDN and PVI and **B** sensitivity analysis after removal of Kiuchi 2017. IV inverse variance, df degrees of freedom.

All studies reported eGFR at baseline, however, only four studies reported eGFR during the follow-up period (Table 4) [17–20]. One of these studies only reported eGFR changes for the PVI + RDN group and was therefore excluded from the meta-analysis [17]. For the purposes of the pooled analysis, the eGFR was compared at the 6-month follow-up period (Fig. 4). The pooled analysis showed a significant MD of +10.22 mL/min/1.73 m^2^ significantly favouring the PVI + RDN group (*p* = 0.0007) (Fig. 4A). Sensitivity analysis was conducted due to the high heterogeneity, and the removal of Kiuchi 2017, led to a decrease in *I*^2^ from 96% to 0 and a stronger significant increase in eGFR following PVI + RDN compared to PVI alone (*P* < 0.00001) (Fig. 4B).

**Table 4:** eGFR at baseline, 6-month, and 12-month of included studies.

Study Author (Year)	Study group	eGFR baseline (mL/min per 1.73 m^2^)	eGFR 6-month (mL/min per 1.73 m^2^)	Mean difference at 6-month (mL/min per 1.73 m^2^)	eGFR 12-month (mL/min per 1.73 m^2^)	Mean difference at 12-month (mL/min per 1.73 m^2^)
**Turagam-HFIB 2 (2021)**^**a**^ [22]	PVI + RDN	>45^a^	NR	__	NR	__
	PVI	>45^a^	NR	__	NR	__
	*P*-value	NS	__	__	__	__
**Turagam-HFIB 1 (2021)**^**a**^ [22]	PVI + RDN	>45^a^	NR	__	NR	__
	PVI	>45^a^	NR	__	NR	__
	*P*-value	NS	__	__	__	__
**Steinberg et al., (2020)** [21]	PVI + RDN	79.0 ± 11.0	NR	__	NR	__
	PVI	76.0 ± 11.0	NR	__	NR	__
	*P*-value	NS	__	__	__	__
**Kiuchi et al., (2018)**^**b**^ [20]	PVI + RDN	69.2 ± 6.70	76.2 ± 7.20	7.00 ± 4.96	81.8 ± 6.8	12.6 ± 4.80
	PVI	66.7 ± 7.70	66.4 ± 8.60	−0.300 ± 5.60	64.8 ± 9.9	−1.90 ± 6.33
	*P*-value	NS	__	<0.0001	__	<0.0001
**Kiuchi et al., (2017)**^**b**^ [19]	PVI + RDN	47.9 ± 6.80	59.0 ± 5.00	11.1 ± 4.52	NR	__
	PVI	50.0 ± 5.40	46.0 ± 5.00	−4.00 ± 3.55	NR	__
	*P*-value	NS	__	__	__	__
**Kiuchi et al., (2016)**^**b**^ [18]	PVI + RDN	59.3 ± 13.3	64.9 ± 13.4	5.60 ± 9.49	65.7 ± 14.0	6.40 ± 9.73
	PVI	60.5 ± 15.9	58.3 ± 14.0	−2.20 ± 10.3	56.6 ± 14.7	−3.90 ± 10.5
	*P*-value	NS	__	NS	__	<0.05
**Pokushalov et al., (2014)** [17]	PVI + RDN	75.5 ± 9.2	80.9 ± 4.3	5.40 ± 6.63	NR	__
	PVI	77.0 ± 8.50	NR	__	__	__
	*P*-value	NS	__	__	__	__

Data are displayed as means and standard deviation (SD).

*PVI* pulmonary vein isolation, *RDN* renal denervation, *eGFR* estimated glomerular filtration rate, *NR* not reported, *NS* not significant.

^a^This study did not report baseline eGFR data but as per the inclusion criteria eGFR of all patients were greater than 45 mL/min per 1.73 m^2^.

### Complications

Five trials reported data on complications following the procedure and during the follow-up period in both the PVI + RDN group and the PVI alone groups [17, 20–22], one reported complications only in the PVI + RDN group [18], and one study failed to specify the group in which the complications occurred and hence the rate could not be estimated for the PVI-alone group [19] (Table 5). HFIB-1 was excluded from the overall rates in both the RDN + PVI and PVI alone groups due to the early termination of study recruitment owing to a high rate of post-RDN renal vascular complications that might be attributed to the use of a non-FDA approved catheter [22]. The overall complication rate between the RDN + PVI and PVI alone group was 6.32% (*n* = 316) and 11.8% (*n* = 245) respectively.

**Table 5:** Complications post-procedures during follow-up period.

Study Author (Year)	Turagam-HFIB 2 (2021) [22]		Turagam-HFIB 1 (2021) [22]^a^		Steinberg et al., (2020) [21]		Kiuchi et al., (2018) [20]		Kiuchi et al., (2017) [19]		Kiuchi et al., (2016) [18]		Pokushalov et al., (2014) [17]	
Study groups	PVI + RDN	PVI	PVI + RDN	PVI	PVI + RDN	PVI	PVI + RDN	PVI + Spironolactone	PVI + RDN	PVI	PVI + RDN	PVI	PVI + RDN	PVI
**Femoral venous vascular events**	2/28	0/22	0	1/17	6/154	4/148	0	0	0	0	0	NR	0	0
**Transient phrenic nerve plasty**	0	0	0	0	1/154	1/148	0	0	0	0	0	NR	0	0
**Cardiac tamponade**	0	0	0	0	0/154	1/148	0	0	0	4/?^b^	0	NR	1/41	0
**Pneumothorax**	0	0	0	0	0/154	1/148	0	0	0	0	0	NR	0	0
**Hospitalization**	0	0	0	0	8/154	18/148	0	0	0	0	0	NR	0	0
**Death**	0	0	0	0	2/154	2/148	0	0	0	0	0	NR	0	0
**Stroke**	0	0	0	1/17	0	0	0	0	0	0	0	NR	0	0
**Renal artery stenosis**	0	0	3/13	0	0	0	0	0	0	0	0	NR	0	0
**Renal artery dissection in procedure**	0	0	3/13	0	0	0	0	0	0	0	0	NR	0	0
**Total**	2/28	0/22	6/13	2/17	17/154	27/148	0	0	0	-	0	NR	1/41	0

Data reported as number of events over sample size.

*PVI* pulmonary vein isolation, *RDN* renal denervation, *NR* not reported.

^a^This study was terminated early due to a high rate of renovascular complications.

^b^Four cardiac tamponade events were reported in this study, but it was not clear which PVI group this occurred in.

## Discussion

We examined the findings from six studies investigating the use of RDN in addition to PVI in the treatment of paroxysmal and/or persistent AF in 711 patients with HTN and AF. The pooled results from these studies showed the following in favor of the RDN and PVI treated group in comparison to PVI alone: (1) A significant reduction in the risk of AF recurrence at follow-up; (2) a significant reduction in SBP at 12-month follow-up; (3) a significant increase in eGFR at 6-month follow-up; (4) a low overall rate of complications in both groups both during the procedures and during the follow-up period.

Currently, the main treatment for HTN is often a combination of lifestyle and drug therapy. First-line agents according to the latest American College of Cardiology and American Heart Association guidelines include angiotensin-converting enzyme inhibitors (ACE-I), thiazide diuretics, and calcium channel blockers [23]. While various drug combinations, doses, and additional agents can be used to reach blood pressure targets, HTN remains the main risk factor for cardiovascular disease including AF and premature death worldwide [24]. Thus, there is a complex pathophysiological mechanism underlying chronic HTN that goes beyond first-line agents addressing the sodium/volume components of the renin-angiotensin-aldosterone system (RAAS).

Studies suggest that HTN can also have neurogenic roots as sympathetic nervous system (SNS) tone is higher in hypertensive patients as opposed to non-hypertensive patients [25]. Increased activity, particularly in the efferent renal nerves, leads to increased stimulation in both renal alpha and beta-adrenoceptors [26]. Stimulation of beta-adrenoreceptors of the juxtaglomerular apparatus increases renin secretion, which ultimately leads to increased systemic vascular resistance and thus arterial pressure [26]. Likewise, increased SNS tone has also been shown to reduce eGFR through the effects of alpha-adrenoceptors on the afferent arterioles [26]. Kidney damage such as due to chronic kidney disease (CKD) or drug-resistant/uncontrolled HTN has been shown to be a driver of increased SNS tone seen in neurogenic HTN [27].

RDN proposes ablation of the renal efferent and afferent nerves to interrupt the communication between the kidney and the autonomic nervous system and thus reduce blood pressure. SYMPLICITY HTN-3, the first of its kind, was a prospective, blinded, sham-controlled trial that included 367 patients and aimed to investigate the effect of RDN on HTN [28]. The trial failed to show any significant blood pressure lowering effects after ablation of the renal nerves [28]. However, it was found that there were many confounders that led to the null effect of SYMPLICITY HTN-3 such as adherence, antihypertensive medications, improper procedural methods, and a lack of operator experience [29]. These among others were addressed in recent trials, leading to significant blood pressure lowering effects of RDN seen in the SPYRAL HTN-ON/OFF MED and RADIANCE-HTN SOLO trials [30–32]. These benefits of RDN were reiterated in a recent meta-analysis of 12 trials (*n* = 1539) and showed that catheter-based RDN is not only effective in the reduction of office blood pressure compared to sham or antihypertensive therapy alone but is also safe for patients [33]. Initially it was thought that RDN would be most efficacious in patients with drug-resistant HTN, but the lessons learned from SYMPLICITY HTN-3 and later trials suggested superior responses of RDN in patient with moderate and neurogenic HTN. Therefore, the ideal candidate for RDN is yet to be identified.

With recent trials supporting the revival of RDN, further findings have also emphasised the potential of its therapeutic uses beyond blood pressure lowering in HTN into AF treatment where HTN is an established risk factor. One of which is seen in a recent post-hoc study of 226 patients from SPYRAL HTN-OFF MED where RDN lowered renin and aldosterone during the follow-up period [34]. For the first time in a human model, the findings of this study established the interaction between RDN, renal sympathetic tone, and HTN [34]. Indeed the improved catheter technology as well as the increase in the sites and frequency of ablation within the renal vasculature has facilitated the utility of RDN beyond just treating HTN [29]. The use of RDN to treat AF can be further supported by various epidemiological studies, one of which (*n* = 1332) significantly showed that a reduction in SBP into lower hypertensive categories reduced the odds of AF recurrence [35]. The present study demonstrated, through a pooled analysis of literature, that in hypertensive patients with AF, when treated with either PVI alone or a combination of PVI and RDN, that the combined treatment group showed a significant mean reduction of SBP by 9.42 mm Hg (*p* = 0.05) and a reduction of DBP by 4.11 mm Hg (*p* = 0.16) at follow-up.

The pathophysiological link between HTN and AF is unclear. However, a review proposed that the link mainly stems from the structural changes associated with HTN, such as LV hypertrophy and LV systolic/diastolic dysfunction which subsequently lead to an increase in left-atrial pressure and fibrosis [36]. Simultaneously, the activation of RAAS further exacerbates those structural modifications and hence leads to electrical remodeling and AF [36]. This hypothesis was evident in our review, where the ablation of the renal nerves, and hence the reduction in sympathetic activation and consequently in RAAS activity, significantly reduced the recurrence of AF by more than 20% (compared to PVI and drug therapy alone), which outlines the involvement of RAAS and HTN in AF development and/or recurrence. Additionally, in one of the studies included in this review, subgroup-analysis of AF recurrence in moderate vs severe resistant hypertensive patients was conducted [17]. Results revealed that in moderate resistant HTN the average blood pressure reduction was −12.5/7.8 mm Hg following RDN and that the rate of AF recurrence was not significantly different between the groups [17]. However, in the severe resistant HTN group, average blood pressure reduction was −29.1/12.2 mm Hg and AF recurrence was significantly lower in the RDN group [17]. In a study by Grassi et al., it was demonstrated that there was a strong positive correlation between sympathetic activity and blood pressure [37]. The study revealed that control subjects had the lowest muscle sympathetic nerve activity compared to those with severe HTN [37]. This suggests that the greater reduction in blood pressure shown in the study by Pokushalov et al. might lead to either a stronger decrease in sympathetic activity or a decrease in sympathetic vascular tone or both, which might have therefore led to the superiority of the decrease in the rate of AF in the severe resistant HTN group [17].

Uncontrolled HTN has been implicated in the development of kidney disease with an average yearly decrease in eGFR of 0.5–2.7 mL/min/1.73 m^2^ [38–40]. The activation of the SNS and RAAS have been identified as the main contributors in the development and progression of renal disease [41]. Therefore, it is clear that AF, HTN and CKD are interlinked and share multiple underlying pathophysiological processes. Interestingly, in one of the included studies in this review, it was noted that patients with CKD had an increased left-atrial volume compared to those without CKD, which therefore contributes strongly to the development of AF [19]. Owing to the hyperactivation of renal sympathetics and RAAS in patients with CKD, the addition of RDN to PVI yielded a stronger decrease in AF recurrence as well as an improvement of multiple structural cardiac parameters including left-atrial volume, LVEF, left-ventricular mass index and left-ventricular end-diastolic diameter [19].

The treatment efforts to dampen RAAS clinically, such as with pharmacological therapy, have failed to yield significant improvements in eGFR and CKD [41]. The progression demonstrated by RDN has led to multiple investigations on the topic and a recent meta-analysis of 11 non-randomized studies was conducted, looking at the effect of RDN in hypertensive patients with CKD [42]. The study mainly concluded that RDN was superior in reducing blood pressure and had no increase in the rate of decline in renal function in patients with CKD. Drawing upon the connection between AF, HTN and CKD, three of the included trials in this review sought to explore the effect of RDN on eGFR and CKD, in an attempt to elucidate the interplay between the outlined mechanisms [18–20]. The pooled analysis showed that RDN significantly improved eGFR compared to PVI alone (MD = 10.2; *p* < 0.001). This may be explained in part by the reduction in sympathetic overdrive following RDN. Kiuchi et al. further noted that more rigorous methods of assessment of renal function should be used in future studies to clearly elucidate the effect of RDN on renal function and CKD [19].

Regarding the safety of RDN, a recent meta-analysis assessing renal function as a safety parameter included 52 quantitative and 14 qualitative studies and concluded that no adverse effects were present following treatment with RDN deeming it as a safe method for use in treatment of HTN [43].

### Clinical implications

The present study, in addition to the aforementioned evidence outlines the critical interplay between HTN, AF and kidney disease, and given the high rate of AF recurrence following PVI alone, the development and consideration of incorporating RDN in addition to current therapeutic strategies to treat both HTN and AF is clinically justified. The pooled analysis further revealed that the use of RDN led to a significant increase in eGFR and hence supported the clinical application of this technology in the context of kidney disease.

Our study supplemented the extensive body of literature on the topic that proved that both procedural and follow-up complications of RDN are low and that the technology has proven to be safe and efficacious with an overall complication rate of 6.32%.

### Limitations

Although multiple reviews have been published on the topic, they failed to account for patients that were concurrently included in multiple studies within the analysis, and have therefore duplicated patients [22, 44–47]. In the 2014 trial by Pokushalov et al., it was stated that the cohort included the 27 patients from the 2012 trial (pilot study) [10, 17]. Furthermore, a trial published in 2017 by Romanov et al. involved the same cohort of patients from the previous 2014 trial by Pokushalov et al., but isolated patients that had cardiac monitor implantation [48]. Indeed, all previously published reviews contain either Pokushalov et al. 2012 and/or 2014 and/or Romanov et al. 2017 and thus incorporate duplicated patients and run the risk of misrepresentation of the data and lack robustness in the true assessment of the impact of RDN on AF and HTN. To this end, this is the first review reporting on the topic with a total of 711 patients as well as including the most recent data following the incorporation of the trials by Turagam et al., [22]. Moreover, our review was the first review on the topic to pool and meta-analyze the effect of RDN on eGFR in patients with HTN and AF.

This review has common limitations to all reviews/analyses as well as specific limitations pertaining to the included studies. Hence, the findings presented are based on the quality of the included studies. Although all studies were prospective, two of those were non-randomized [18, 19]. Furthermore, the reliability of the reported results depends on the consistency in inclusion criteria and the methods employed by the studies, including baseline characteristics, underlying comorbidities, definition of HTN (i.e., HTN cut-off values) and AF, number of antihypertensive and anti-arrhythmic drugs, catheterization and ablation methods, differences in follow-up times as well as methods to assess the outcomes during follow-up; all of which may have varied between the studies and hence might have affected the reliability and introduced heterogeneity in our results. The heterogeneity was combatted by conducting a sensitivity analysis. Finally, it is also worth noting that one of the included trials (HFIB-1) was terminated early due to an increase in renovascular complications, which may be owing to the use of a non-FDA approved catheter and hence caution should be exercised when assessing pooled outcomes that included the mentioned study [22].

## Conclusion and future directions

This review demonstrated that the introduction of RDN to PVI in hypertensive patients with AF is more efficacious and superior to using PVI alone in treating AF. RDN + PVI was also shown to reduce SBP more significantly in patients with resistant HTN as well improve eGFR outcomes. Moreover, analysis of the safety of the technique proved it to be safe and hence the introduction of RDN to PVI should be considered clinically in patients with AF. Larger and longer-term trials are required to substantiate these findings including those that utilise sham-controls to improve robustness of the assessed outcomes. Future trials should also assess the effect of the autonomic reduction of blood pressure on AF and hence examine whether the effect of RDN on AF is dependent solely on autonomic reduction or if there is a mechanism independent of blood pressure that contributes to the improvement in AF.

### Summary

#### What is known about topic?

- There is an established interaction between renal denervation, renal sympathetic tone, and hypertension. The recent introduction of endovascular catchers to lower moderate/resistant hypertension has yielded promising results due to its ability to dampen the renin-angiotensin-aldosterone axis.
- The success rate of pulmonary vein isolation in reducing atrial fibrillation is limited (20–50%). The significant morbidity associated with atrial fibrillation and the complex interaction of atrial fibrillation and hypertension has prompted the investigation of the additive benefit of renal denervation, with preliminary results of multiple trials demonstrating the superiority of such method in improving outcomes compared to conventional therapy.

#### What this study adds?

- The pooled analysis demonstrates that combined renal denervation and pulmonary vein isolation reduces atrial fibrillation recurrence compared to pulmonary vein isolation alone. This supports the inclusion of renal denervation in the management of atrial fibrillation. The overall safety of the technique has proven it to be safe and efficacious.
- The analysis outlines the critical interplay between atrial fibrillation, hypertension, and kidney function, and demonstrates the significant blood pressure lowering effect of renal denervation.
- Renal denervation was also shown to have a significant effect on kidney function via an improvement in estimated glomerular filtration rate, which is hypothesized to be due to the dampening effects on the renin-angiotensin-aldosterone axis.

## Supplementary information

Supplementary Table S1 and Figure S1

## References

[1] Lippi G, Sanchis-Gomar F, Cervellin G (2021). Global epidemiology of atrial fibrillation: an increasing epidemic and public health challenge. Int J Stroke.

[2] Morillo CA, Banerjee A, Perel P, Wood D, Jouven X (2017). Atrial fibrillation: the current epidemic. J Geriatr Cardiol.

[3] Benjamin EJ, Muntner P, Alonso A, Bittencourt MS, Callaway CW, Carson AP (2019). Heart Disease and Stroke Statistics-2019 Update: A Report From the American Heart Association. Circulation.

[4] Lloyd-Jones D, Adams RJ, Brown TM, Carnethon M, Dai S, De Simone G, et al. Heart Disease and Stroke Statistics—2010 Update. Circulation. 2010;121. 10.1161/CIRCULATIONAHA.109.192667.

[5] Esler HK, PA S, MP S, RE S, M B (2010). Renal sympathetic denervation in patients with treatment-resistant hypertension (The Symplicity HTN-2 Trial): a randomised controlled trial. Lancet.

[6] Silverwatch J, Marti KE, Phan MT, Amin H, Roman YM, Pasupuleti V (2021). Renal Denervation for Uncontrolled and Resistant Hypertension: Systematic Review and Network Meta-Analysis of Randomized Trials. J Clin Med.

[7] Mark DB, Anstrom KJ, Sheng S, Piccini JP, Baloch KN, Monahan KH (2019). Effect of Catheter Ablation vs Medical Therapy on Quality of Life Among Patients With Atrial Fibrillation. JAMA.

[8] Calkins H, Hindricks G, Cappato R, Kim Y-H, Saad EB, Aguinaga L (2018). 2017 HRS/EHRA/ECAS/APHRS/SOLAECE expert consensus statement on catheter and surgical ablation of atrial fibrillation: Executive summary. EP Eur.

[9] Packer DL, Mark DB, Robb RA, Monahan KH, Bahnson TD, Poole JE (2019). Effect of Catheter Ablation vs Antiarrhythmic Drug Therapy on Mortality, Stroke, Bleeding, and Cardiac Arrest Among Patients With Atrial Fibrillation. JAMA.

[10] Pokushalov E, Romanov A, Corbucci G, Artyomenko S, Baranova V, Turov A (2012). A Randomized Comparison of Pulmonary Vein Isolation With Versus Without Concomitant Renal Artery Denervation in Patients With Refractory Symptomatic Atrial Fibrillation and Resistant Hypertension. J Am Coll Cardiol.

[11] Kung J, Chiappelli F, Cajulis OO, Avezova R, Kossan G, Chew L (2010). From Systematic Reviews to Clinical Recommendations for Evidence-Based Health Care: Validation of Revised Assessment of Multiple Systematic Reviews (R-AMSTAR) for Grading of Clinical Relevance. Open Dent J.

[12] Moher D, Liberati A, Tetzlaff J, Altman DG, Altman D, Antes G (2009). Preferred reporting items for systematic reviews and meta-analyses: The PRISMA statement (Chinese edition). J Chin Integr Med.

[13] Slim K, Nini E, Forestier D, Kwiatkowski F, Panis Y, Chipponi J (2003). Methodological index for non-randomized studies (MINORS): development and validation of a new instrument. ANZ J Surg.

[14] McHugh ML (2012). Interrater reliability: the kappa statistic. Biochem Med.

[15] Higgins JPT, Thompson SG, Deeks JJ, Altman DG (2003). Measuring inconsistency in meta-analyses. BMJ.

[16] Higgins JP, Green S. Cochrane Handbook for Systematic Reviews of Interventions: Cochrane Book Series. 2008. 10.1002/9780470712184.

[17] Pokushalov E, Romanov A, Katritsis DG, Artyomenko S, Bayramova S, Losik D (2014). Renal denervation for improving outcomes of catheter ablation in patients with atrial fibrillation and hypertension: Early experience. Hear Rhythm.

[18] Kiuchi ChenS, e Silva GR, Paz LMR, Kiuchi T, de Paula Filho AG (2016). Pulmonary vein isolation alone and combined with renal sympathetic denervation in chronic kidney disease patients with refractory atrial fibrillation. Kidney Res Clin Pr.

[19] Kiuchi ChenS, e Silva GR, Rodrigues Paz LM, Kiuchi T, de Paula Filho AG (2017). The addition of renal sympathetic denervation to pulmonary vein isolation reduces recurrence of paroxysmal atrial fibrillation in chronic kidney disease patients. J Inter Card Electrophysiol.

[20] Kiuchi ChenS, Hoye NA, Pürerfellner H (2018). Pulmonary vein isolation combined with spironolactone or renal sympathetic denervation in patients with chronic kidney disease, uncontrolled hypertension, paroxysmal atrial fibrillation, and a pacemaker. J Inter Card Electrophysiol.

[21] Steinberg JS, Shabanov V, Ponomarev D, Losik D, Ivanickiy E, Kropotkin E (2020). Effect of Renal Denervation and Catheter Ablation vs Catheter Ablation Alone on Atrial Fibrillation Recurrence Among Patients With Paroxysmal Atrial Fibrillation and Hypertension: The ERADICATE-AF Randomized Clinical Trial. JAMA.

[22] Turagam MK, Whang W, Miller MA, Neuzil P, Aryana A, Romanov A (2021). Renal Sympathetic Denervation as Upstream Therapy During Atrial Fibrillation Ablation. JACC Clin Electrophysiol.

[23] Whelton PK, Carey RM, Aronow WS, Casey DE, Collins KJ, Dennison Himmelfarb C (2018). 2017 ACC/AHA/AAPA/ABC/ACPM/AGS/APhA/ASH/ASPC/NMA/PCNA Guideline for the Prevention, Detection, Evaluation, and Management of High Blood Pressure in Adults: A Report of the American College of Cardiology/American Heart Association Task Force on Clinical Pr. Hypertension.

[24] Mills KT, Stefanescu A, He J (2020). The global epidemiology of hypertension. Nat Rev Nephrol.

[25] Esler M (2000). The sympathetic system and hypertension*1. Am J Hypertens.

[26] Johns EJ, Abdulla MH (2013). Renal nerves in blood pressure regulation. Curr Opin Nephrol Hypertens.

[27] Schlaich MP, Socratous F, Hennebry S, Eikelis N, Lambert EA, Straznicky N (2009). Sympathetic Activation in Chronic Renal Failure: Figure 1. J Am Soc Nephrol.

[28] Bakris GL, Townsend RR, Flack JM, Brar S, Cohen SA, D’Agostino R (2015). 12-Month Blood Pressure Results of Catheter-Based Renal Artery Denervation for Resistant Hypertension. J Am Coll Cardiol.

[29] Sharif F (2018). Renal artery denervation: a lot done and more to do. EuroIntervention.

[30] Townsend RR, Mahfoud F, Kandzari DE, Kario K, Pocock S, Weber MA (2017). Catheter-based renal denervation in patients with uncontrolled hypertension in the absence of antihypertensive medications (SPYRAL HTN-OFF MED): a randomised, sham-controlled, proof-of-concept trial. Lancet.

[31] Kandzari DE, Böhm M, Mahfoud F, Townsend RR, Weber MA, Pocock S (2018). Effect of renal denervation on blood pressure in the presence of antihypertensive drugs: 6-month efficacy and safety results from the SPYRAL HTN-ON MED proof-of-concept randomised trial. Lancet.

[32] Azizi M, Schmieder RE, Mahfoud F, Weber MA, Daemen J, Davies J (2018). Endovascular ultrasound renal denervation to treat hypertension (RADIANCE-HTN SOLO): a multicentre, international, single-blind, randomised, sham-controlled trial. Lancet.

[33] Cheng X, Zhang D, Luo S, Qin S (2019). Effect of Catheter-Based Renal Denervation on Uncontrolled Hypertension: A Systematic Review and Meta-analysis. Mayo Clin Proc.

[34] Mahfoud F, Townsend RR, Kandzari DE, Kario K, Schmieder RE, Tsioufis K (2021). Changes in Plasma Renin Activity After Renal Artery Sympathetic Denervation. J Am Coll Cardiol.

[35] Thomas MC, Dublin S, Kaplan RC, Glazer NL, Lumley T, Longstreth WT (2008). Blood Pressure Control and Risk of Incident Atrial Fibrillation. Am J Hypertens.

[36] Lau Y-F, Yiu K-H, Siu C-W, Tse H-F (2012). Hypertension and atrial fibrillation: epidemiology, pathophysiology and therapeutic implications. J Hum Hypertens.

[37] Grassi G, Cattaneo BM, Seravalle G, Lanfranchi A, Mancia G (1998). Baroreflex Control of Sympathetic Nerve Activity in Essential and Secondary Hypertension. Hypertension.

[38] Chowdhury EK, Langham RG, Ademi Z, Owen A, Krum H, Wing LMH (2015). Rate of Change in Renal Function and Mortality in Elderly Treated Hypertensive Patients. Clin J Am Soc Nephrol.

[39] Zoppini G, Targher G, Chonchol M, Ortalda V, Negri C, Stoico V (2012). Predictors of Estimated GFR Decline in Patients with Type 2 Diabetes and Preserved Kidney Function. Clin J Am Soc Nephrol.

[40] Vupputuri S, Batuman V, Muntner P, Bazzano LA, Lefante JJ, Whelton PK (2003). Effect of Blood Pressure on Early Decline in Kidney Function Among Hypertensive Men. Hypertension.

[41] Converse RL, Jacobsen TN, Toto RD, Jost CMT, Cosentino F, Fouad-Tarazi F (1992). Sympathetic Overactivity in Patients with Chronic Renal Failure. N. Engl J Med.

[42] Xia M, Liu T, Chen D, Huang Y (2021). Efficacy and safety of renal denervation for hypertension in patients with chronic kidney disease: a meta-analysis. Int J Hyperth.

[43] Sanders MF, Reitsma JB, Morpey M, Gremmels H, Bots ML, Pisano A (2017). Renal safety of catheter-based renal denervation: systematic review and meta-analysis. Nephrol Dial Transpl.

[44] Atti V, Turagam MK, Garg J, Lakkireddy D (2019). Renal sympathetic denervation improves clinical outcomes in patients undergoing catheter ablation for atrial fibrillation and history of hypertension: A meta‐analysis. J Cardiovasc Electrophysiol.

[45] Ukena C, Becker N, Pavlicek V, Millenaar D, Ewen S, Linz D (2020). Catheter-based renal denervation as adjunct to pulmonary vein isolation for treatment of atrial fibrillation: a systematic review and meta-analysis. J Hypertens.

[46] Pranata R, Vania R, Raharjo SB (2020). Efficacy and safety of renal denervation in addition to pulmonary vein isolation for atrial fibrillation and hypertension—Systematic review and meta‐analysis of randomized controlled trials. J Arrhythmia.

[47] Mujer MT, Al‐Abcha A, Saleh Y, Nerusu LA, Boumegouas M, Herzallah K (2020). Effect of combined renal denervation and pulmonary vein isolation in atrial fibrillation recurrence in hypertensive patients: A meta‐analysis. Pacing Clin Electrophysiol.

[48] Romanov A, Pokushalov E, Ponomarev D, Strelnikov A, Shabanov V, Losik D (2017). Pulmonary vein isolation with concomitant renal artery denervation is associated with reduction in both arterial blood pressure and atrial fibrillation burden: Data from implantable cardiac monitor. Cardiovasc Ther.

