# Supplementary material for: Renal denervation for atrial fibrillation: a comprehensive updated systematic review and meta-analysis
Source: J Hum Hypertens. 2022 Jan 29;36(10):887–97. doi: 10.1038/s41371-022-00658-0 (PMC9553644; doi:10.1038/s41371-022-00658-0)
Supplement: Supplementary file 1 — Supplementary Table S1 and Figure S1 [file 41371_2022_658_MOESM1_ESM.pdf]

This supplementary file includes the risk of bias assessment for the included studies including the MINORS assessment for non-Randomized studies and the Cochrane Risk of Bias Tool for Randomized studies

Table S1: MINORS score assessment for Non-Randomized studies

Study Author (Year)	Kiuchi et al., (2017)[19]
**A stated aim of the study**	2
**Inclusion of consecutive patients**	2
**Prospective collection of data**	2
**Endpoint appropriate to the study aim**	2
**Unbiased evaluation of endpoints**	1
**Follow-up period appropriate to the major endpoint**	2
**Loss to follow up not exceeding 5%**	1
**A control group having the gold standard intervention**	2
**Contemporary groups**	2
**Baseline equivalence of groups**	2
**Prospective calculation of the sample size**	1
**Statistical analyses adapted to the study design**	2
**MINORS score**	21

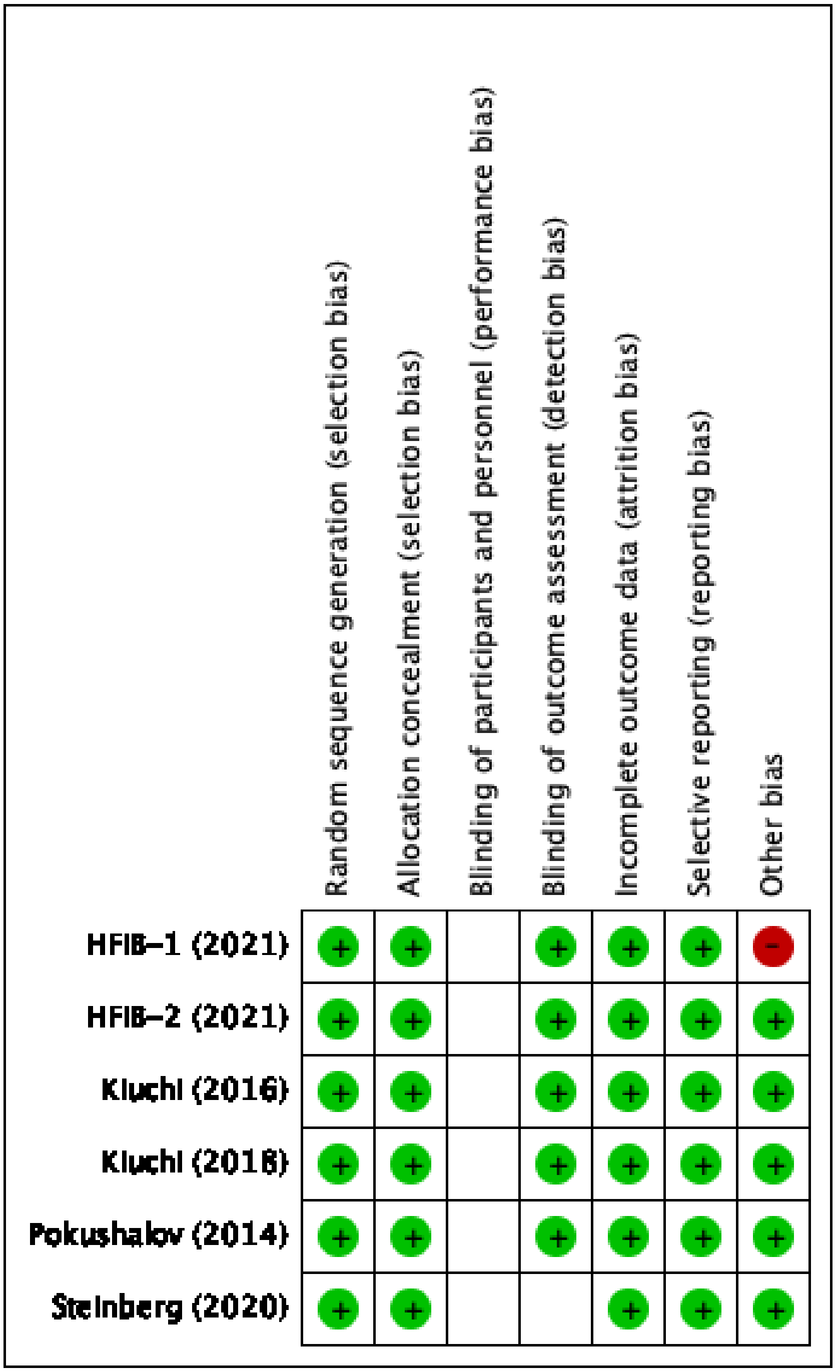

Figure S1: Cochrane Risk of Bias Tool for Randomized studies. Green/Positive indicates low risk of bias, Red/Negative indicates a high risk of bias and empty space indicates unclear risk of bias.
